# Nerve Stretching in Tetanus

**Published:** 1877-01-20

**Authors:** 


### NERVE STRETCHING IN TETANUS.

G. W. Callender, Surgeon to St. Bartholomew's Hospital, in *The Lancet*, advocates the treatment of tetanus by nerve stretching. He writes:

I am glad to hear that, quite recently, M. Verneuil has had under his care, in La Pitie, a case which he will, I hope, shortly publish.

A man had suffered from a severe crush of the hand, and, following this, showed the symptoms of tetanus. Mr. Verneuil exposed the median nerve at the elbow, and the ulner at the wrist, and proceeded to exercise traction on them. The patient recovered completely.

I hope this note may lead to a further trial of this method of treatment. The operation is not a severe one. The nerve is exposed and stretched, when freed from its surroundings, by traction with an ordinary vulsellum, from its central connections. No harm is likely to be sustained as a consequence. There is now abundant evidence, in the cases reported by Billroth, Nussbaum, and myself, of the tolerance with which nerves submit to forcible stretching, so far as the after-performance of their functions is concerned. In view of the unsatisfactory results of the treatment of traumatic tetanus as at present conducted, there is full justification for the performance of the operation as, at least, a last resource, although I should myself advocate its trial, as in the case under the care of M. Verneuil, as soon as the signs of the disease are distinctly recognized.

